# Synthesis and structures of ruthenium–NHC complexes and their catalysis in hydrogen transfer reaction

**DOI:** 10.3762/bjoc.11.194

**Published:** 2015-09-30

**Authors:** Chao Chen, Chunxin Lu, Qing Zheng, Shengliang Ni, Min Zhang, Wanzhi Chen

**Affiliations:** 1 College of Life Sciences, Huzhou University, Huzhou 313000, China; 2 College of Biological, Chemical Sciences and Engineering, Jiaxing University, Jiaxing 314001, China; 3 Department of Chemistry, Zhejiang University, Hangzhou 310007, China

**Keywords:** N-heterocyclic carbene, ruthenium, transfer hydrogenation

## Abstract

Ruthenium complexes [Ru(L1)_2_(CH_3_CN)_2_](PF_6_)_2_ (**1**), [RuL1(CH_3_CN)_4_](PF_6_)_2_ (**2**) and [RuL2(CH_3_CN)_3_](PF_6_)_2_ (**3**) (L1= 3-methyl-1-(pyrimidine-2-yl)imidazolylidene, L2 = 1,3-bis(pyridin-2-ylmethyl)benzimidazolylidene) were obtained through a transmetallation reaction of the corresponding nickel–NHC complexes with [Ru(*p*-cymene)_2_Cl_2_]_2_ in refluxing acetonitrile solution. The crystal structures of three complexes determined by X-ray analyses show that the central Ru(II) atoms are coordinated by pyrimidine- or pyridine-functionalized N-heterocyclic carbene and acetonitrile ligands displaying the typical octahedral geometry. The reaction of [RuL1(CH_3_CN)_4_](PF_6_)_2_ with triphenylphosphine and 1,10-phenanthroline resulted in the substitution of one and two coordinated acetonitrile ligands and afforded [RuL1(PPh_3_)(CH_3_CN)_3_](PF_6_)_2_ (**4**) and [RuL1(phen)(CH_3_CN)_2_](PF_6_)_2_ (**5**), respectively. The molecular structures of the complexes **4** and **5** were also studied by X-ray diffraction analysis. These ruthenium complexes have proven to be efficient catalysts for transfer hydrogenation of various ketones.

## Introduction

N-Heterocyclic carbenes (NHCs) have been recognized as a class of strong donating ligands which can stabilize various metal complexes of catalytic importance. Transition metal complexes bearing NHCs are more stable to air, moisture, heat, and tolerant toward oxidation compared to phosphine ligands [1–7]. Among NHCs, functionalized NHC ligands have been extensively studied in recent years because of their intriguing structural diversities and potential applications in coordination chemistry and homogenous catalysis. NHC ligands containing additional phosphine, nitrogen, oxygen, and sulfur donating groups [8–16] have been reported.

In the family of metal complexes supported by functionalized NHCs, ruthenium complexes have long been a research focus on various applications such as catalysis and photochemistry [17–26]. However, the majority of such ruthenium complexes often contain coordinated aromatic carbocycles [27–29]. In contrast, only a few examples Ru(II) complexes of functionalized NHCs containing easily dissociating acetonitrile ligands have been studied [30–32]. We have reported the synthesis of some pyridine- and phenanthrolin-functionalized Ru(II)–NHC complexes containing acetonitrile ligands [33–34]. The most notable example is the acetonitrile-coordinated dinuclear Ru(II)–NHC complex derived from 3,6-bis(*N*-(pyridylmethyl)imidazolylidenyl)pyridazine, which is a very efficient catalyst for the oxidation of alkenes [35]. In continuation of our studies on functionalized Ru(II)–NHC complexes containing acetonitrile ligands, we herein report the synthesis and characterization of three pyrimidine- and pyridine-functionalized NHC–ruthenium complexes containing two, four, and three acetonitrile ligands, respectively. These complexes show good catalytic activity in the transfer hydrogenation of ketones. The reaction of acetonitrile-coordinated Ru–NHC complex **2** with other donors such as triphenylphosphine and 1,10-phenanthroline was also studied.

## Results and Discussion

### Synthesis and characterization of [Ru(L1)_2_(CH_3_CN)_2_](PF_6_)_2_ (**1**), [RuL1(CH_3_CN)_4_](PF_6_)_2_ (**2**) and [RuL2(CH_3_CN)_3_](PF_6_)_2_ (**3**)

**Scheme 1:**
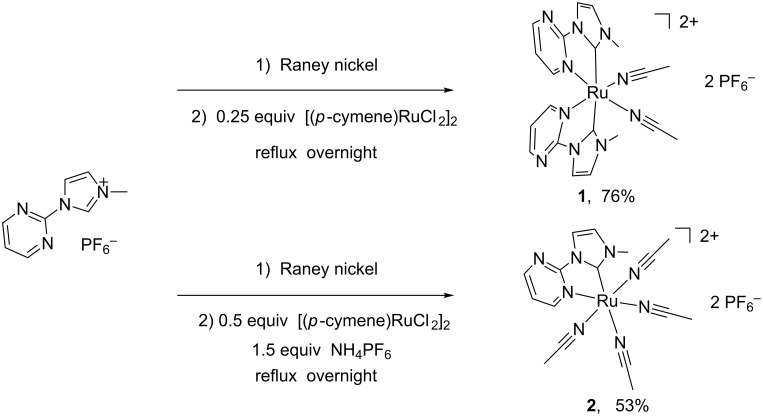
Synthesis of complexes **1** and **2**.

The ruthenium–NHC complexes **1** and **2** were synthesized by using the corresponding nickel–NHC complexes as the carbene transfer agent [36]. The reaction of imidazolium salt HL1(PF_6_) (L1 = 3-methyl-1-(pyrimidine-2-yl)imidazolylidene) with Raney nickel afforded the nickel–NHC complexes which were not isolated [30]. The subsequent reaction of the generated nickel–NHC complexes with a quarter equivalent of [Ru(*p*-cymene)Cl_2_]_2_ in refluxing acetonitrile solution afforded bis-NHC complex [Ru(L1)_2_(CH_3_CN)_2_](PF_6_)_2_ (**1**) in a yield of 76% (Scheme 1). When a half equivalent of [Ru(*p*-cymene)Cl_2_]_2_ and an excess of NH_4_PF_6_ were employed under the same conditions, the reaction afforded the mono-NHC complex [RuL1(CH_3_CN)_4_](PF_6_)_2_ (**2**) in 53% yield. It is worth noting that most of the structurally characterized acetonitrile complexes are obtained through the reaction of halides with silver complexes (AgPF_6_ or AgBF_4_) in acetonitrile solution [20]. The reaction in refluxing acetonitrile is more convenient than the above mentioned procedure. The formulations of complexes **1** and **2** were first characterized by NMR measurements and further confirmed by elemental analysis and X-ray diffraction. In the ^1^H NMR spectra of complexes **1** and **2**, disappearance of the resonances assigned to the imidazolium acidic CH and *p*-cymene protons were observed. The acetonitrile protons of complex **1** were found at 2.41 ppm as a singlet. However, the protons of acetonitrile ligands of complex **2** were found at 2.52, 2.12, and 1.96 ppm as three singlets. This illustrates that the three acetonitrile ligands in complex **2** are magnetic unequivalent. The ^13^C NMR spectra of **1** and **2** exhibit resonance signals at 193.1 and 193.0 ppm ascribed to the carbenic carbons.

**Figure 1:**
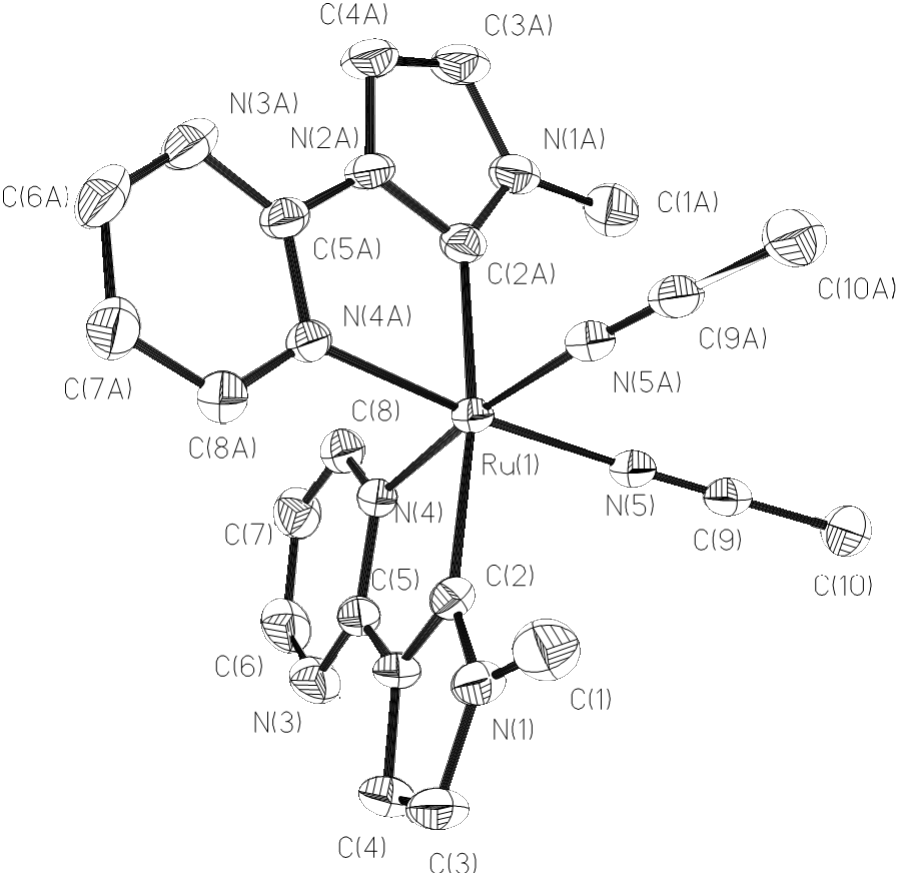
Structural view of **1** showing 30% thermal ellipsoids. All hydrogen atoms and PF_6_^−^ were omitted for clarity. Selected bond lengths (Å) and angles (deg): Ru(1)–N(5) 2.033(4), Ru(1)–C(2) 2.066(5), Ru(1)–N(4) 2.081(4), N(5)#1–Ru(1)–N(5) 83.9(2), N(5)–Ru(1)–C(2) 87.87(16), C(2)#1–Ru(1)–C(2) 171.2(3), N(5)–Ru(1)–N(4) 91.12(16), C(2)#1–Ru(1)–N(4) 95.88(17), N(5)–Ru(1)–N(4)#1 174.20(14). Symmetry code: #1 −x, y, −z+1/2.

The ruthenium–NHC complexes **1** and **2** are stable in air and under light irradiation. Single crystals suitable for X-ray diffraction could be obtained by slow diffusion of Et_2_O into CH_3_CN solutions and the detailed structure of **1** is depicted in Figure 1. In complex **1**, the central ruthenium ion is hexacoordinated by two bidentate NHC ligands and two acetonitrile ligands in an octahedral geometry. One NHC ligand, one acetonitrile ligand and one carbon atom of the other NHC ligand occupy the equatorial plane in which two carbon atoms of two NHC ligands are mutually *trans*-arranged. The remaining acetonitrile ligand and one nitrogen atom of the NHC ligand lie on the axial positions. The angles (N–Ru–N) of adjacent nitrogen atoms and Ru(II) ion are in the range of 83.9 to 94.0°. The Ru–C distance (2.066 Å) is consistent with the reported values in known Ru–NHC complexes [17–29]. The Ru–N_pyrimidine_ distance (2.081 Å) is slightly longer than Ru–N_acetonitrile_ (2.033 Å).

**Figure 2:**
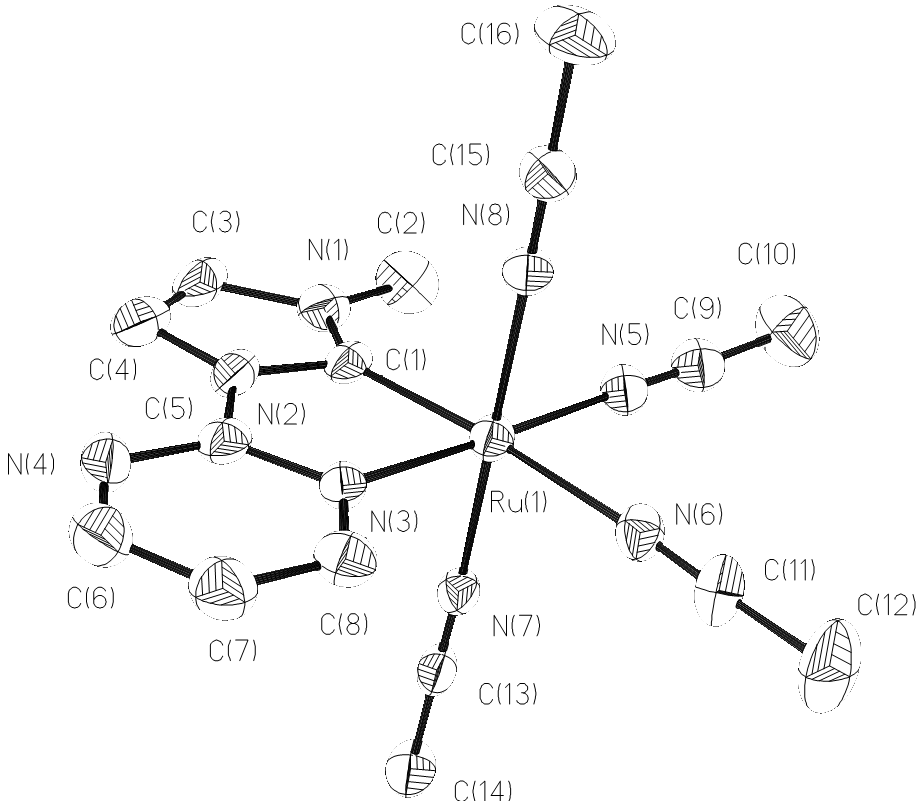
Structural view of **2** showing 30% thermal ellipsoids. All hydrogen atoms and PF_6_^−^ were omitted for clarity. Selected bond lengths (Å) and angles (deg): Ru(1)–C(2) 1.989(7), Ru(1)–N(5) 2.023(5), Ru(1)–N(8) 2.027(5), Ru(1)–N(7) 2.033(6), Ru(1)–N(4) 2.064(5), Ru(1)–N(6) 2.113(6), C(2)–Ru(1)–N(5) 88.3(2), C(2)–Ru(1)–N(8) 91.2(2), N(5)–Ru(1)–N(8) 179.3(2), C(2)–Ru(1)–N(7) 99.8(3).

The cationic structure of **2** is shown in Figure 2. The central Ru(II) ion is surrounded by one pyrimidine-functionalized NHC ligand and four acetonitrile ligands also in a typical octahedral geometry. The Ru ion lies on a twofold axis. The bidentate NHC ligand and two *cis*-arranged acetonitrile molecules form a Ru(L1)(CH_3_CN)_2_ plane, whereas the other two acetonitrile molecules occupy the axial positions. The bond length of Ru–C_NHC_ is 1.989 Å, which is slightly shorter than those found in Ru–NHC complexes [12–18] and in complex **1**. The bond distance of Ru–N_acetonitrile_ (2.113 Å) at the *trans*-position of the carbene ligand is longer than the other three Ru–N_acetonitrile_ bonds (2.023–2.033 Å) and the Ru–N_pyrimidine_ (2.064 Å).

**Scheme 2:**
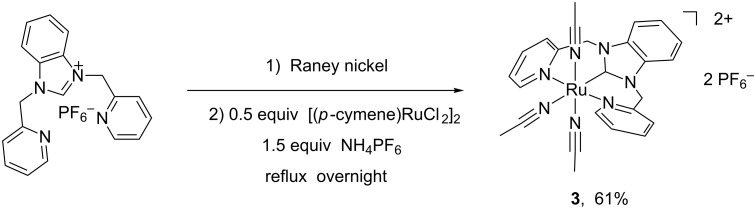
Synthesis of **3**.

**Figure 3:**
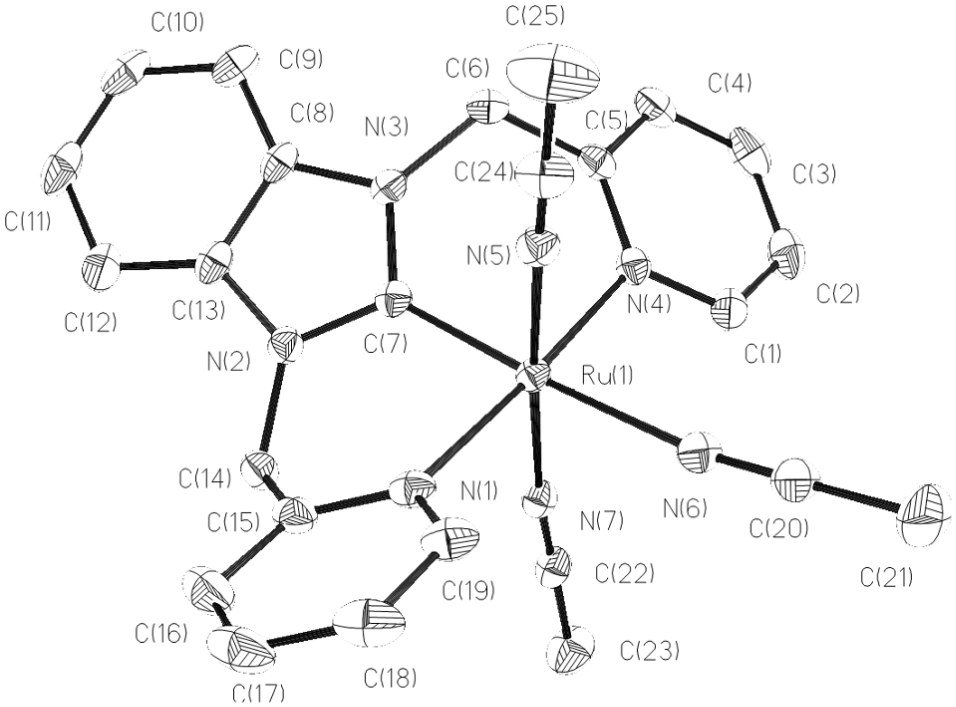
Structural view of **3** showing 50% thermal ellipsoids. All hydrogen atoms and PF_6_^−^ were omitted for clarity. Selected bond lengths (Å) and angles (deg): Ru(1)–C(7) 1.947(3), Ru(1)–N(7) 2.028(2), Ru(1)–N(5) 2.030(2), Ru(1)–N(4) 2.104(2), Ru(1)–N(1) 2.105(2), Ru(1)–N(6) 2.130(2), C(7)–Ru(1)–N(7) 94.33(10), C(7)–Ru(1)–N(5) 90.55(10), N(7)–Ru(1)–N(5) 174.99(9), C(7)–Ru(1)–N(4) 87.72(10).

Similarly, the reaction of the in situ generated nickel–NHC complex from imidazolium salt HL2(PF_6_) (L2 = 1,3-bis(pyridin-2-ylmethyl)benzimidazolylidene) with a half equivalent of [Ru(*p*-cymene)Cl_2_]_2_ and an excess of NH_4_PF_6_ in a refluxing acetonitrile solution afforded the tri-acetonitrile coordinated Ru(II)–NHC complex [RuL2(CH_3_CN)_3_](PF_6_)_2_ (**3**) in a yield of 61% (Scheme 2). The formation of **3** was also confirmed by the ^1^H NMR and ^13^C NMR spectra. The ^1^H NMR spectrum of **3** shows characteristic resonance signals due to the pyridyl, methylene, benzimidazolylidene and acetonitrile groups. The absence of a benzimidazole acidic C2-H proton illustrates the formation of the Ru–C bond. The acetonitrile protons appear at 2.35 and 2.08 ppm as two singlets. The ^13^C NMR spectrum of **3** exhibits a resonance peak at 190 ppm, which is ascribed to the carbenic carbon atom. Complex **3** has been further identified by X-ray crystallography and the cationic structure of molecular **3** is depicted in Figure 3. The ruthenium ion is coordinated by a tridentate pincer NHC ligand and three acetonitrile ligands also in an octahedral geometry. The symmetrical pincer-type NCN ligand and an acetonitrile ligand occupy the equatorial plane and the remaining two acetonitrile ligands are located at the axial positions. The N–Ru–N angles of the three acetonitrile ligands and the Ru(II) ion are 86.03, 89.12 and 174.99°, respectively. Similar to complex **2**, the bond distance of Ru–N_acetonitrile_ (2.130 Å) at the *trans*-position of the carbene ligand is slightly longer than the other bond distances of Ru–N_acetonitrile_ (2.030 and 2.028 Å) and the Ru–C (1.947 Å) is shorter than that of many known Ru–C_carbene_ distances [17–29].

### Catalytic transfer hydrogenation reaction

Ruthenium–NHC complexes are known to be efficient catalysts for transfer hydrogenation reactions [23,37–39]. The ruthenium–NHC complexes presented above are stabilized by strong Ru–carbene bonds and contain 2–4 easily dissociating acetonitrile molecules, and are thus ideal catalysts. We tested their catalytic activities for transfer hydrogenation of ketones. Firstly, acetophenone was selected as the model substrate to evaluate the catalytic activities of complexes **1**–**3**. The standard experiment was carried out at 80 °C with varied Ru loadings from 1 to 0.01 mol % and the results are summarized in Table 1. The reaction profiles show that acetophenone could be reduced to 1-phenylethanol in 89–99% yield within 0.5 h using 1 mol % of the Ru catalysts (Table 1, entries 1, 5 and 9). When the amount of catalysts is decreased to 0.1 mol %, the corresponding conversion still reached 79–89% (Table 1, entries 2, 6 and 10). 1-Phenylethanol could also be obtained in excellent yields using 0.1 mol % and 0.01 mol % Ru catalysts when the reaction time was extended to 1 and 3 h, respectively (Table 1, entries 3, 7, 11 and 4, 8, 12). At catalyst loadings of 0.01 mol %, TOF of **1**–**3** are 3000, 3233, and 3200 h^−1^ for transfer hydrogenation of acetophenone which are nearly identical to that of [Ru(^Me^CC^meth^)_2_(CH_3_CN)_2_](BF_4_)_2_ (^Me^CC^meth^ = 1,1'-dimethyl-3,3'-methylene-diimidazol-2,2'-diylidene) [40]. Ruthenium picolyl–NHC complex [(*η*^5^-C_5_Me_5_)-Ru(L)(CH_3_CN)][PF_6_] (L = 3-methyl-1-(2-picolyl)imidazol-2-ylidene) is so far one of the most efficient catalyst for transfer hydrogenation of acetophenone which gave 1-phenylethanol in a conversion of 93% with a catalyst loading of 0.1 mol % [20,41]. When the same amount of complexes **1**–**3** was used, the reaction gave 1-phenylethanol in 89%, 99% and 99% yields, respectively. These data illustrate that complexes **1**–**3** are all quite active catalysts for transfer hydrogenation reactions. It seems that complexes **2** and **3** are a bit better than **1** for this transformation. The *trans*-effect of carbene ligand may promote the substitution of *trans*-positioned acetonitrile ligand by other substrates in the catalytic reaction.

**Table 1:** Catalytic activities of **1**–**3** in transfer hydrogenation of acetophenone.^a^

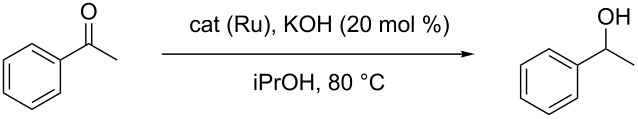					

Entry	Catalyst	Catalyst (mol %)	Time (h)	Yield (%)^b^	TON/TOF (h^−1^)

1	**1**	1	0.5	89	89/172
2		0.1	0.5	79	790/1580
3		0.1	1	92	920/920
4		0.01	3	90	9000/3000

5	**2**	1	0.5	99	99/198
6		0.1	0.5	86	860/1720
7		0.1	1	99	990/990
8		0.01	3	97	9700/3233

9	**3**	1	0.5	99	99/198
10		0.1	0.5	89	890/1780
11		0.1	1	99	990/990
12		0.01	3	96	9600/3200

^a^Conditions: acetophenone (1.00 mmol), KOH (20 mol %), and catalyst (1–0.01 mol %) in 3 mL of iPrOH at 80 °C. ^b^The yields of products were detected by GC.

Since complexes **2** and **3** are found to be the efficient catalysts for transfer hydrogenation of acetophenone, we further explored their catalytic potential in the reduction of other aromatic and aliphatic ketones. The reaction conditions are similar as those described in the transfer hydrogenation of acetophenone and 0.1 mol % of Ru catalyst is utilized. The obtained results are given in Table 2. Complexes **2** and **3** are found to be very active in transfer hydrogenation of cyclohexanone, and cyclohexanol are almost quantitatively yielded within 0.5 h (Table 2, entries 1 and 2). The catalyst systems are also found to be good for the reduction of aromatic ketones bearing electron-withdrawing substituents (Table 2, entries 3–8) and electron-donating groups (Table 2, entries 9 and 10), and the target product could be obtained in excellent yields (90–99%). Bulkier aromatic ketone benzophenone is also tested in this reaction with 92% and 94% conversion after 3 h (Table 2, entries 11 and 12). In addition, it is worth mentioning that the two ruthenium complexes exhibited a high tolerance towards sulfur species, 2-acetylthiophene is efficiently hydrogenated (Table 2, entries 13 and 14) with an increased reaction time of 3 h.

**Table 2:** Transfer hydrogenation using complexes **2** and **3**.^a^

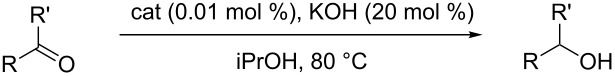				

Entry	Substrate	Catalyst	Time (h)	Yield (%)^b^

1	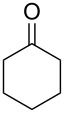	2	0.5	99
	
2		3	0.5	99

3	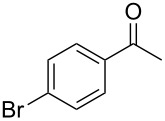	2	1	99
	
4		3	1	98

5	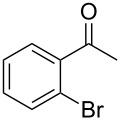	2	1	99
	
6		3	1	97

7	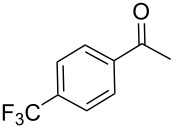	2	1	96
	
8		3	1	90

9	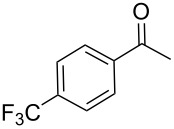	2	1	93
	
10		3	1	92

11	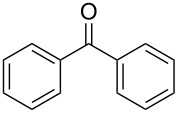	2	3	92
	
12		3	3	94

13	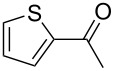	2	3	83
	
14		3	3	80

^a^Conditions: substrate (1.00 mmol), KOH (20 mol %), catalyst (0.1 mol %) in 3 mL of iPrOH at 80 °C. ^b^The yields of products were detected by GC.

### Reactions of tetra-acetonitrile Ru(II)–NHC complex **2** with triphenylphosphine and 1,10-phenanthroline

**Scheme 3:**
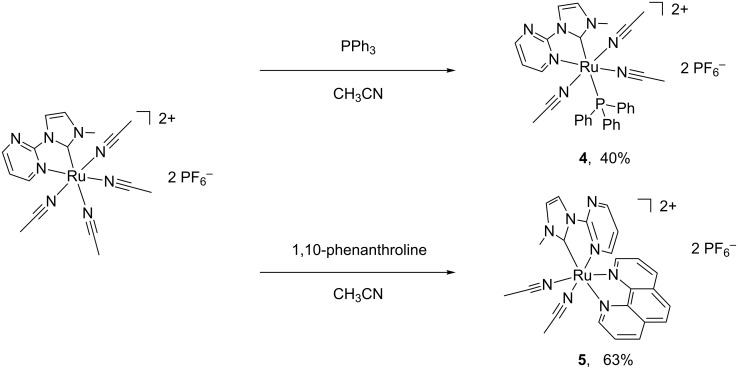
Synthesis of complexes **4** and **5**.

The coordinated acetonitrile ligands could be easily replaced by various *N*- and *P*-donors [22]. The reactions of the acetonitrile-coordinated Ru–NHC complexes with other ligands were studied. The reaction of complex **2** with an excess of triphenylphosphine and 1,10-phenanthroline in heat acetonitrile solution afforded **4** and **5**, respectively. Even excess triphenylphosphine and 1,10-phenanthroline were used, only one and two coordinated acetonitrile ligands were substituted in complexes **4** and **5**. Crystallization by slow diffusion of diethyl ether into their acetonitrile solutions gave **4** as a yellow solid in 40% yield and **5** as an orange yellow solid in 63% yield (Scheme 3). The yields of complexes **4** and **5** are relatively lower than complexes **1**–**3**, but still in the normal range as compared with the similar reaction [33]. In the ^1^H NMR of **4**, singlets at 2.14 and 2.07 ppm are ascribed to three CH_3_CN ligands, and the rest peaks are belonged to NHC and triphenylphosphine ligand. ^1^H NMR investigation of **5** suggests that complex **5** contains one NHC ligand, one phenanthroline ligand and two acetonitrile ligands. The CH_3_CN protons of **5** are founded at 2.53 and 2.28 ppm. In the ^13^C NMR, the carbene carbons of complexes **4** and **5** are found at 190 and 200 ppm, respectively.

**Figure 4:**
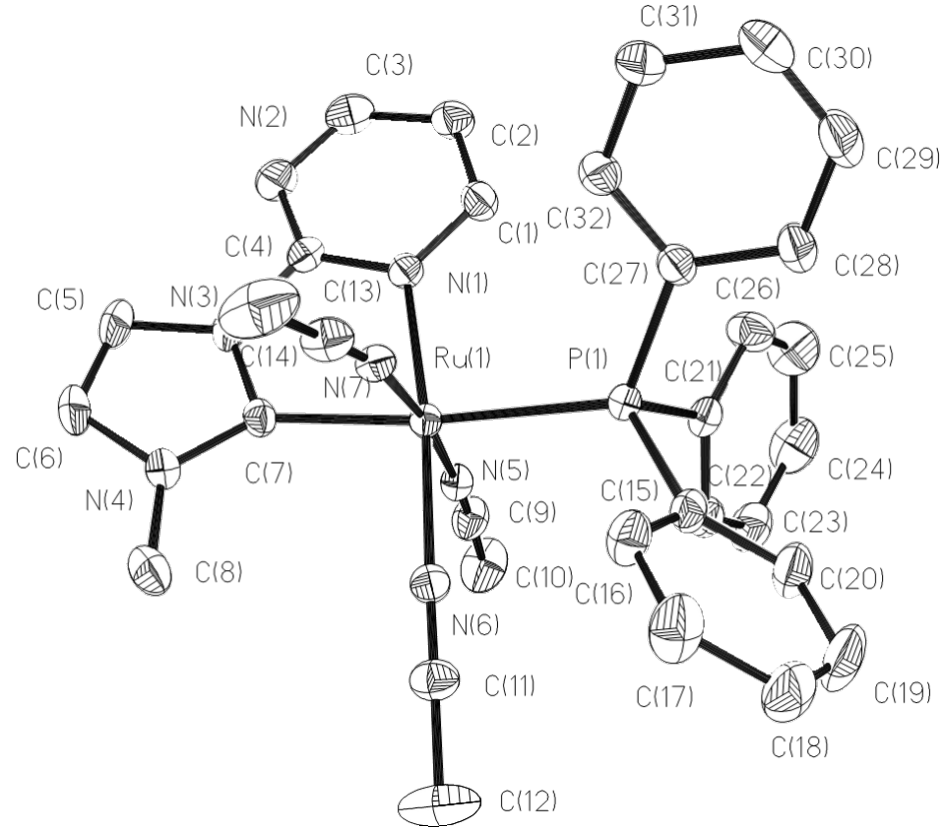
Structural view of **4** showing 30% thermal ellipsoids. All hydrogen atoms and PF_6_^−^ were omitted for clarity. Selected bond lengths (Å) and angles (deg): Ru(1)–N(5) 2.012(4), Ru(1)–N(7) 2.020(4), Ru(1)–N(6) 2.025(4), Ru(1)–C(7) 2.039(4), Ru(1)–N(1) 2.120(4), Ru(1)–P(1) 2.4080(11), N(5)–Ru(1)–N(7) 173.83(16), N(5)–Ru(1)–N(6) 87.01(16), N(7)–Ru(1)–N(6) 89.42(15), N(5)–Ru(1)–C(7) 92.27(17), N(5)–Ru(1)–P(1) 92.91(11), C(7)–Ru(1)–P(1) 173.12(16).

**Figure 5:**
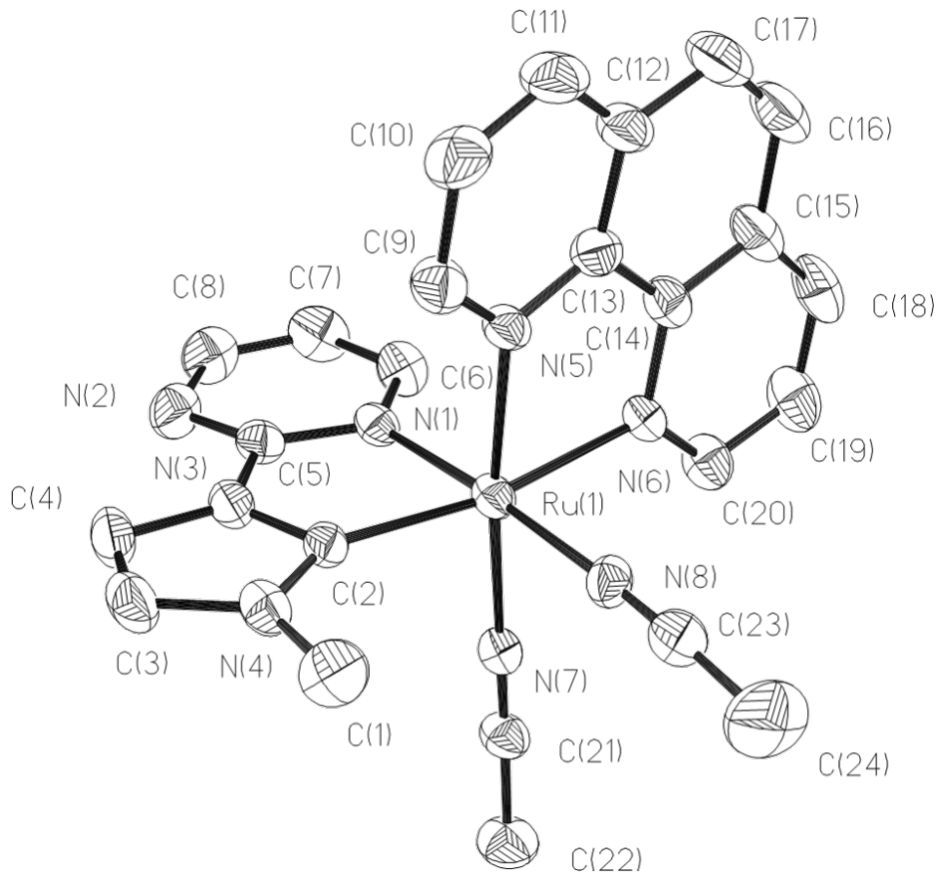
Structural view of **5** showing 30% thermal ellipsoids. All hydrogen atoms and PF_6_^−^ were omitted for clarity. Selected bond lengths (Å) and angles (deg): Ru(1)–C(2) 2.007(5), Ru(1)–N(8) 2.022(4) Ru(1)–N(7) 2.049(4), Ru(1)–N(5) 2.063(4), Ru(1)–N(1) 2.077(4), Ru(1)–N(6) 2.126(4), C(2)–Ru(1)–N(8) 99.06(18), C(2)–Ru(1)–N(7) 91.10(17), N(8)–Ru(1)–N(7) 87.76(16), C(2)–Ru(1)–N(5) 95.03(17), N(8)–Ru(1)–N(5) 90.05(16), N(7)–Ru(1)–N(5) 173.74(16). Symmetry code: #1 x, −y+3/2, z.

The structures of **4** and **5** determined by X-ray diffraction analysis are shown in Figure 4 and Figure 5. In the cationic structure of **4**, the acetonitrile ligand at the *trans*-position of the NHC is substituted by a triphenylphosphine ligand. The CNPN atoms form the equatorial plane. The other two acetonitrile ligands are still *trans*-arranged at the axial positions. The P–Ru–N angles of three acetonitrile ligands and pyrimidine are 92.91, 92.06, 88.91, and 98.34°. The Ru–C bond distances being 2.039 Å is slightly longer than those of **2** and **3**, but similar to complex **1**. The Ru–P bond distance is 2.4080 Å, which are no difference from those of reported Ru(II) complexes [3–4]. In complex **5**, the central Ru ion is coordinated by one NHC ligand, one 1,10-phenanthroline ligand and two acetonitrile molecules. The NHC ligand, one acetonitrile ligand and one nitrogen atom of phenanthroline occupy the equatorial plane in which the carbon atom of NHC ligand is *trans* to the nitrogen atom of phenanthroline with the C(2)–Ru(1)–N(6) angle of 169.08°, the acetonitrile molecule is *trans* to the pyrimidine group with the N(8)–Ru(1)–N(1) angle of 176.42°. The rest coordination nitrogen atoms of acetonitrile and phenanthroline lie on the axial positions with the N(7)–Ru(1)–N(5) angle of 173.74°.

## Conclusion

In summary, Ru–NHC complexes bearing pyrimidine- and pyridine-functionalized NHC ligands have been prepared through a carbene transfer reaction using nickel–NHC as the carbene source. Their structures have been definitely determined by X-ray crystallography. The catalytic behavior of di-, tetra- and tri-acetonitrile-coordinated ruthenium complexes in transfer hydrogenation reactions was studied. These ruthenium complexes were found to be highly efficient catalysts for transfer hydrogenation of ketones. The catalytic properties of the ruthenium complexes in other organic transformation will be further studied.

## Experimental

All chemicals were obtained from commercial suppliers in reagent grade quality and were used as received. HL1PF_6_ and HL2PF_6_ were synthesized according to the reported method [42–43]. ^1^H and ^13^C NMR spectra were recorded on a Bruker Avance-400 (400 MHz) spectrometer operating at 400 MHz for ^1^H and at 100 MHz for ^13^C. Chemical shifts (δ) were expressed in ppm downfield to TMS at δ = 0 ppm and coupling constants (*J*) were expressed in Hz. Elemental analyses were performed by a Flash EA 1112 ThermoFinnigan analyzer.

**Synthesis of [Ru(L1)_2_(CH_3_CN)_2_](PF_6_)_2_ (1).** A mixture of HL1(PF_6_) (306 mg, 1.0 mmol), excess Raney nickel (500 mg) in 10 mL MeCN was stirred at 80 °C for 24 h. After it was cooled to room temperature, the solution was filtered through Celite. Then [Ru(*p*-cymene)Cl_2_]_2_ (153 mg, 0.25 mmol) was added to the solution and stirred at reflux for 12 h. After filtration through a plug of Celite, the mixture was concentrated and poured into Et_2_O (30 mL) to precipitate the product. Compound **1** was obtained as a yellow solid. Yield: 307 mg, 76%. Anal. calcd for C_20_H_22_F_12_N_10_P_2_Ru: C, 30.27; H, 2.79; N, 17.65; found: C, 30.19; H, 2.82; N, 17.55; ^1^H NMR (400 MHz, DMSO-*d*_6_) δ 8.77 (d, *J* = 4.8 Hz, C_4_H_3_N_2_, 2H), 8.31 (d, *J* = 2.0 Hz, C_3_H_2_N_2_, 2H), 8.09 (d, *J* = 4.8 Hz, C_4_H_3_N_2_, 2H), 7.90 (d, *J* = 2.0 Hz, C_4_H_3_N_2_, 2H), 7.27 (t, *J* = 4.8 Hz, C_4_H_3_N_2_, 2H), 4.17 (s, CH_3_, 3H), 2.41 (s, CH_3_CN, 6H); ^13^C NMR (100 MHz, DMSO-*d*_6_) δ 193.1 (Ru-*C*), 166.2, 159.8, 158.7, 128.6, 127.0, 120.0, 117.9, 37.7, 4.17.

**Synthesis of [RuL1(CH_3_CN)_4_](PF_6_)_2_ (2).** A mixture of HL1(PF_6_) (153 mg, 0.5 mmol), excess Raney nickel (300 mg) in 10 mL MeCN was stirred at 80 °C for 24 h. After it was cooled to room temperature, the solution was filtered through Celite. Then [Ru(*p*-cymene)Cl_2_]_2_ (153 mg, 0.25 mmol) and NH_4_PF_6_ (163 mg, 1.0 mmol) was added to the filtrate and stirred at reflux for 12 h. The mixture was filtered through Celite to remove precipitated NiCl_2_ and all volatiles were evaporated under reduced pressure. The residue was washed with water and dried in vacuo. The yellow residue was dissolved in MeCN and concentrated to about 2 mL. The addition of Et_2_O induced precipitation of the product as a yellow solid. Yield: 190 mg, 53%. Anal. calcd for C_16_H_20_F_12_N_8_P_2_Ru: C, 26.86; H, 2.82; N, 15.66; found: C, 26.70; H, 2.90; N, 15.58; ^1^H NMR (400 MHz, DMSO-*d*_6_) δ 9.12 (d, *J* = 4.8 Hz, C_4_H_3_N_2_, 1H), 8.85 (d, *J* = 4.8 Hz, C_4_H_3_N_2_, 1H), 8.00 (d, *J* = 2.4 Hz, C_3_H_2_N_2_, 1H), 7.48 (t, *J* = 2.4 Hz, C_4_H_3_N_2_, 1H), 7.36 (d, *J* = 2.4 Hz, C_3_H_2_N_2_, 1H), 4.04 (s, CH_3_, 3H), 2.52, (s, CH_3_CN, 3H), 2.12, (s, CH_3_CN, 6H), 1.96, (s, CH_3_CN, 3H); ^13^C NMR (100 MHz, DMSO-*d*_6_) δ 193.0 (Ru-*C*), 180.9, 166.6, 158.4, 158.3, 157.4, 128.7, 125.7, 125.6, 117.7, 116.2, 35.8, 2.64, 2.20, 1.77.

**Synthesis of [RuL2(CH_3_CN)_3_](PF_6_)_2_ (3).** According to the same procedure as described for **2**, complex **3** was obtained as a yellow soild. Yield: 249 mg, 61%. Anal. calcd for C_29_H_31_F_12_N_9_P_2_Ru ([RuL2(CH_3_CN)_3_](PF_6_)_2_)·2CH_3_CN: C, 38.85; H, 3.48; N, 14.06; found: C, 38.70; H, 3.60; N, 14.08; ^1^H NMR (400 MHz, DMSO-*d*_6_) δ 8.90 (d, *J* = 4.4 Hz, C_5_H_4_N, 2H), 8.11 (t, *J* = 6.4 Hz, C_5_H_4_N, 2H), 7.95–7.92 (m, C_6_H_4_, 4H), 7.64 (t, *J* = 5.2 Hz, C_5_H_4_N, 2H), 7.41–7.40 (m, C_5_H_4_N, 2H), 5.85 (s, CH_2_, 4H), 2.35, (s, CH_3_CN, 6H), 2.08 (s, CH_3_CN, 3H); ^13^C NMR (100 MHz, DMSO-*d*_6_) δ 192.0 (Ru-*C*), 154.1, 148.2, 140.5, 134.7, 125.5, 125.3, 125.0, 117.2, 116.6, 111.4, 50.5, 2.80, 2.15.

**Synthesis of [RuL1(PPh_3_)(CH_3_CN)_3_](PF_6_)_2_ (4).** A mixture of **2** (142 mg, 0.2 mmol) and triphenylphosphine (262 mg, 1.0 mmol) in 5 mL CH_3_CN was stirred at 80 °C for 6 h. Then the mixture was filtered through Celite and all volatiles were evaporated under reduced pressure. The residue was washed with ethyl acetate and dried in vacuo. The yellow residue was dissolved in CH_3_CN and crystallization by slow diffusion of Et_2_O into the CH_3_CN solution gave **4** as yellow solid. Yield: 75 mg, 40%. Anal. calcd for C_32_H_32_F_12_N_7_P_3_Ru: C, 41.04; H, 3.44; N, 10.47; found: C, 41.10; H, 3.40; N, 10.58; ^1^H NMR (DMSO-*d*_6_) δ 8.99 (s, 1H), 8.46 (s, 1H), 8.38 (s, 1H), 7.87 (s, 1H), 7.57(s, 13H), 7.42 (s, 2H), 7.25 (s, 1H), 4.11 (s, 3H), 2.14 (s, 6H), 2.07 (s, 3H); ^13^C NMR (DMSO-*d*_6_) δ 192.2 (Ru-C), 183.8, 182.8, 163.4, 158.6, 157.9, 132.0, 131.9, 131.7, 131.5, 129.0, 128.7, 127.9, 127.6, 127.5, 127.4, 127.2, 127.1, 126.3, 125.5, 117.9, 116.4, 116.3, 35.8, 2.15, 1.72.

**Synthesis of [RuL1(Phen)(CH_3_CN)_2_](PF_6_)_2_ (5).** A mixture of **2** (142 mg, 0.2 mmol) and 1,10-phenanthroline·1H_2_O (198 mg, 1.0 mmol) in 5 mL CH_3_CN was stirred at 80 °C for 6 h. Then the mixture was filtered through Celite to afford a yellow solution. Crystallization by slow diffusion of Et_2_O into the CH_3_CN solution gave **5** as an orange yellow solid. Yield: 103 mg, 63%. Anal. calcd for C_24_H_22_F_12_N_8_P_2_Ru: C, 35.43; H, 2.73; N, 13.77; found: C, 35.50; H, 2.90; N, 13.80; ^1^H NMR (DMSO-*d*_6_) δ 9.77 (dd, *J* = 1.2 and 4.0 Hz, 1H), 9.08 (dd, *J* = 0.8 and 6.4 Hz, 1H), 8.75 (dd, *J* = 1.6 and 4.0 Hz, 1H), 8.44–8.38 (m, 3H), 8.32–8.29 (m, 2H), 7.94 (d, *J* = 1.6 Hz, 1H), 7.73 (dd, *J* = 4.4 and 6.4 Hz, 1H), 7.68 (dd, *J* = 1.6 and 6.4 Hz, 1H), 7.11 (dd, *J* = 4.0 and 4.4 Hz, 1H), 4.23 (s, 3H), 4.22 (s, 3H), 2.53, 2.28 (s, CH_3_CN, each 3H); ^13^C NMR (DMSO-*d*_6_) δ 192.3 (Ru-C), 161.9, 159.9, 159.0, 157.5, 152.5, 148.0, 146.6, 138.8, 137.4, 130.9, 130.6, 128.3, 128.2, 127.4, 127.2, 127.1, 126.4, 119.5, 118.5, 37.1, 4.56, 3.83, 1.62.

### Typical procedure for catalytic transfer hydrogenation reaction

The ketone (1.0 mmol), KOH (0.2 mmol) and 2 mL of iPrOH were placed in a Schlenk tube. Anisole (0.25 mmol) was added as an internal GC standard. The mixture was heated at 80 °C and then catalyst solution (0.01 mmol, 0.001 mmol, or 0.0001 mol of ruthenium complexes in iPrOH (1 mL) was injected. Aliquots (0.2 mL) were taken at fixed time intervals, quenched with 1 mL of H_2_O and extracted with 3 mL of Et_2_O. The product yields were determined by GC analysis.

### X-ray diffraction analysis

Single-crystal X-ray diffraction data were collected at 298(2) K on a Siemens Smart-CCD area-detector diffractometer with a MoKα radiation (λ = 0.71073 Å) by using a ω-2θ scan mode. Unit-cell dimensions were obtained with least-squares refinement. Data collection and reduction were performed using the Oxford Diffraction CrysAlisPro software [44]. All structures were solved by direct methods, and the non-hydrogen atoms were subjected to anisotropic refinement by full-matrix least squares on *F*^2^ using the SHELXTXL package [45]. Hydrogen atom positions for all of the structures were calculated and allowed to ride on their respective C atoms with C–H distances of 0.93–0.97 Å and *U*_iso_(H) = −1.2–1.5*U*_eq_(C). Details of the X-ray experiments and crystals data are summarized in Table 3.

**Table 3:** Crystallographic data for complexes **1**–**5**.

	**1**	**2**	**3**·2CH_3_CN	**4**·CH_3_CN	**5**

CCDC number	1407422	1407423	1407424	1407425	1407426
Formula	C_20_H_22_F_12_N_10_P_2_ Ru	C_16_H_20_F_12_N_8_P_2_ Ru	C_29_H_31_F_12_N_9_P_2_ Ru	C_34_H_35_F_12_N_8_P_3_ Ru	C_24_H_22_F_12_N_8_P_2_ Ru
*Fw*	793.49	715.41	896.64	977.68	813.51
crystal system	Monoclinic	Monoclinic	Triclinic	Triclinic	Monoclinic,
space group	*C*2/*c*	*P*2/*n*	*P*−*1*	*P−1*	*P*2_1_/*m*
*a*, Å	23.240(3)	11.2914(5)	11.4695(12)	9.9130(16)	10.9570(8)
*b*, Å	10.3410(5)	12.7244(6)	13.1322(14)	12.665(2)	22.2567(16)
*c*, Å	16.060(4)	21.4357(11)	13.7721(14)	2 18.222(3)	16.8706(11)
α, deg	90	90	97.7010	90	90
β, deg	130.19(3)	102.469(4)	103.2130	90	97.384(6)
γ, deg	90	90	94.0570	66.96	90
*V*, Å^3^	2948.4(8)	3007.2(2)	1990.1(4)	2105.2(6)	4080.1(5)
*Z*	4	4	2	2	4
*D*_calcd_, Mg/m^3^	1.788	1.580	1.496	1.542	1.324
Reflections collected	5571	10931	15882	7390	15951
Reflections independent (*R*_int_)	2597 (0.0289)	5299 (0.0492)	7002 (0.0129)	7390 (0.0000)	7385 (0.0278)
Goodness-of-fit on *F*^2^	1.059	1.064	1.053	1.050	1.083
*R* (*I* > 2σ*I*)	0.0539, 0.1465	0.0712, 0.2121	0.0373, 0.0973	0.0418, 0.1020	0.0604, 0.1788
*R* (all data)	0.0617, 0.1558	0.0913, 0.2322	0.0389, 0.0984	0.0455, 0.1049	0.0794, 0.1904

## Supporting Information

Supporting Information File:

File 1 X-ray crystallographic data CCDC 1407422–1407426.
